# Gliding Swifts Attain Laminar Flow over Rough Wings

**DOI:** 10.1371/journal.pone.0099901

**Published:** 2014-06-25

**Authors:** David Lentink, Roeland de Kat

**Affiliations:** 1 Department of Mechanical Engineering, Stanford University, Stanford, California, United States of America; 2 Experimental Zoology Group, Wageningen University, Wageningen, The Netherlands; 3 Department of Aerospace Engineering, Delft University of Technology, Delft, The Netherlands; University of Sussex, United Kingdom

## Abstract

Swifts are among the most aerodynamically refined gliding birds. However, the overlapping vanes and protruding shafts of their primary feathers make swift wings remarkably rough for their size. Wing roughness height is 1–2% of chord length on the upper surface—10,000 times rougher than sailplane wings. Sailplanes depend on extreme wing smoothness to increase the area of laminar flow on the wing surface and minimize drag for extended glides. To understand why the swift does not rely on smooth wings, we used a stethoscope to map laminar flow over preserved wings in a low-turbulence wind tunnel. By combining laminar area, lift, and drag measurements, we show that average area of laminar flow on swift wings is 69% (n = 3; *std* 13%) of their total area during glides that maximize flight distance and duration—similar to high-performance sailplanes. Our aerodynamic analysis indicates that swifts attain laminar flow over their rough wings because their wing size is comparable to the distance the air travels (after a roughness-induced perturbation) before it transitions from laminar to turbulent. To interpret the function of swift wing roughness, we simulated its effect on smooth model wings using physical models. This manipulation shows that laminar flow is reduced and drag increased at high speeds. At the speeds at which swifts cruise, however, swift-like roughness prolongs laminar flow and reduces drag. This feature gives small birds with rudimentary wings an edge during the evolution of glide performance.

## Introduction

**Figure 1:**
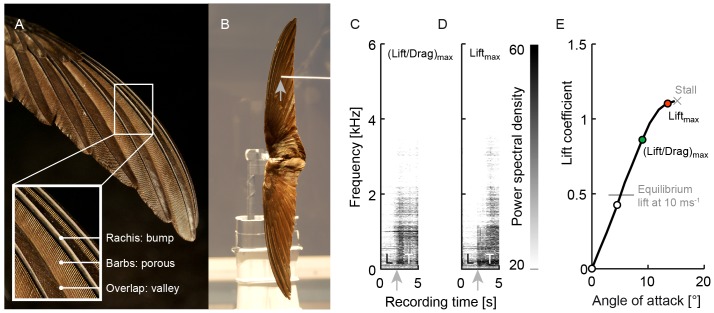
Detection of boundary layer transition to turbulence on swift wings with an amplified stethoscope[30]. (A) Similarly to other birds, swift hand wings are built up by overlapping primary feathers that make the upper surface corrugated. (B) When broad-spectrum turbulent noise was detected with the stethoscope, a photo of the transition location (grey arrow) was made (Movie S1). (C) Spectrogram of a snippet of Movie S1 showing laminar (L) – turbulent (T) transition over the hand wing at maximal lift-drag ratio. (D) Spectrogram of transition at maximum lift (Movie S2). (E) Lift – angle of attack curve corresponding to transition measurements in (C) at 9° and in (D) at 13.5°, when the wing is partially stalled. White and colored circles are transition measurement points; grey line indicates equilibrium lift during straight glide at 10 ms^−1^; cross indicates wing stall.

Feathers are a key adaptation that distinguishes bird wings [1]–[3] from wings of other flying animals such as bats [4] and insects [5]. Articulated overlapping feathers uniquely enable birds to morph their wings continuously from fully extended to completely folded, to optimize the shape for high aerodynamic performance [6]. Wing morphing enables common swifts (*Apus apus L.*) to glide up to 60% further and 100% longer [7]. Among birds, swifts stand out because they spend almost their entire lives aloft: swifts not only migrate, they also hunt insects, scoop water, mate, and even roost in flight. Their extremely aerial lifestyle drives swifts to conserve energy by alternating flapping bouts with many low-drag glides [7]. These glides resemble those of sailplanes, because soaring birds and pilots have similar performance objectives when they exploit thermals and updrafts in the atmosphere to fly more effectively cross-country [8]. Sailplanes have extremely smooth wings and fuselages in order not to perturb the laminar airflow over the surface and thereby minimize drag. The wing surface architecture of swifts and sailplanes are, however, different as night and day. The overlapping centimeter-scale feathers consist of a central rachis with an anterior and posterior vane of barbs. The barbs branch out into distal barbules with micrometer-scale hooklets that connect to the flanges of proximal barbules – a function similar to that of Velcro [3]. As a result, the wing surface is porous at the micro scale, and leaks a small amount of air from the lower to the upper surface [9], potentially affecting friction drag [10]. At the macro scale the protruding feather rachis, together with the valleys created by the overlap between primary feathers [11], make the hand wing of swifts corrugated with a roughness height of 1–2% (Fig. 1A–C). In stark contrast, well-designed sailplane wings have a roughness height of 0.0001% or less in order to prolong laminar airflow for exceptionally long glides [12]. It is unclear how swifts, which are among the most aerodynamically refined birds [7], [13], [14], glide so well with hand wings that are a factor 10,000 rougher than high-performance sailplane wings.

The aerodynamic impact of feather roughness depends on the roughness height relative to the thickness of the layer of air that sticks to, and flows over, the wing – the boundary layer [10], [15], [16]. When boundary layer thickness and roughness height are similar, the air can become disturbed to the point that it transitions from laminar to turbulent flow [10], [16]. The boundary layer thickness 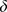 depends on the chord length 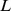 and speed 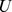
of the wing via Reynolds number. The wing's Reynolds number *Re* = 68,000×*U*×*L* represents the ratio of inertial to viscous forces in the boundary layer [16] (in which 68,000 is the inverse of the kinematic viscosity of air). *Re*≈24,000 is close to the swift's most efficient glide speed (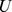
≈10 ms^−1^; 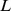
≈0.036 m; [7], [14]). Using a flat plate approximation (δ/L = 5*Re* ^−0.5^≈0.03; [10]), we estimate that boundary layer thickness is 3% of the chord length at the trailing edge. Because surface roughness of the swift hand wing is 1–2% it may disturb the airflow to the point that it becomes turbulent [10], [16]. Surface roughness elements that force the flow to become turbulent are called ‘turbulators’ [16]. Experiments with model pigeon wings (*Re* = 60,000–100,000) have shown that turbulators made of sandpaper can increase lift and decrease drag by reducing laminar flow separation [1]. Roughness effects have also been suggested to influence force measurements on real versus model hummingbird wings [17]. A study of laminar-turbulent transition on the rough upper surface of a non-porous 3D-printed hummingbird wing, at 10° angle of attack, suggests that the boundary layer flow is transitional and turbulent at 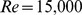 [18].

The detailed shape of a turbulator matters [19]–[21]. If we ignore the amplifying effect of periodicity [20], the ridges on swift wings resemble simple strip turbulators, small rectangular surface protrusions, which are more effective than sandpaper [20]. The effectiveness of a turbulator can be determined more precisely by calculating its roughness Reynolds number, defined as *Re*_k_ = *Re*×*k*/*L*, where *k* is the roughness height [22]. The minimum values of 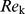
for which strip turbulators initiate turbulence range from 80 to 170, as measured at *Re* = 100,000 for various roughness heights, strip locations, and airfoils [23]. This critical 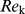
range was confirmed by [24] with a value of *Re*_k_ = 110 at *Re* = 100,000 and by [25], who found *Re*_k_ = 70–130 for *Re* = 150,000. The effectiveness of these thin strips is demonstrated by model airplanes soaring in the atmosphere under conditions similar to swifts [26]. Swifts typically glide at a Reynolds number range of 12,000–77,000 [7], resulting in *Re*_k_>120–770 for *k*/*L*>1%. Therefore, the swift hand wing should have sufficient aerodynamic roughness to initiate turbulence at all biologically relevant airspeeds. The extent of laminar flow in the thin boundary layer over feathered bird wings is, however, unknown. An understanding of how feather roughness affects laminar flow at low Reynolds numbers is also applicable to hair-covered mammal wings with protruding arm, hand, and finger bones [4], [27], and to scale-covered flying reptile bodies [28].

## Materials and Methods

### Wing roughness measurements

**Figure 2:**
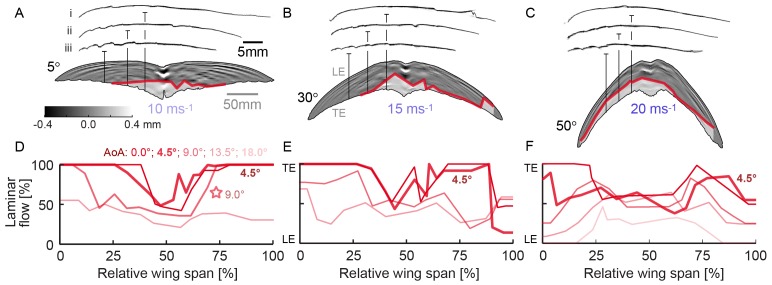
Spanwise transition occurs aft of prominent feather roughness at maximal glide performance. (A, B, C) The primary feathers of the hand wing give a roughness of 1–2% chord length (iii) distal (red line: transition; translucent area: turbulent flow). The arm wing is smoothened by greater primary coverts at the wrist (ii) and greater secondary coverts and marginal coverts proximal (i). Transition occurs well behind the most prominent feather roughness on the hand wing at 4.5° angle of attack. This angle maximizes glide distance and flight duration at the speed and sweep combinations in *a*, *b* and *c*. Both the scanned surface contour (raw) and the fitted surface contour (60% transparent overlay) are shown (D, E, F). For low angles of attack, transition is most prominent near the centerline, due to imperfections in the glued joint between the left and right wing and corresponding coverts. Laminar flow decreases with increasing angle of attack. The star indicates the distribution at 9° and 10 ms^−1^. This distribution is closest to 8.5° and 8 ms^−1^, at which the swift flies furthest and longest according to our lift and drag measurements [7]. Asymmetries in the distribution reflect shape asymmetry. (LE: leading edge; TE: trailing edge).

The wings were separated from naturally deceased birds, frozen, and freeze dried, after which matched pairs were glued together to form a continuous wing surface [7]. Of 15 wing pairs we selected three with particularly well-preserved wing and feather condition: fully extended at 5° sweep angle and swept back at 30° and 50° (n = 3 birds, by combining all three wing sweeps). Whereas the overall condition of the wings was very good, the epoxy joint at the centerline introduced some roughness and ruffled coverts at the wing root. We measured the chord-wise position and height of roughness on the upper wing surface (Fig. 2) of swift wings with 5°, 30° and 50° sweep using a custom 3D laser-line scan setup. The laser sheet was created perpendicular to the wing surface with a green laser pen (532 nm wavelength) and a two-lens system: A negative cylindrical lens to create a sheet, and a perpendicular positive spherical lens to create a thin waist. The projected line was imaged with a camera (Nikon J1; 10–30 mm Nikkor lens) at approx. 30° off-normal to avoid occlusion. We calibrated the scan setup using a metal ruler with known vertical and horizontal distances (approx. 65 mm field of view) located at the beam waist (resolution: 65 mm/3872 pixels ≈0.017 mm/pixel). To reconstruct the surface contour of prepared wings, the projected line was photographed at each spanwise station (Fig. 2A–C, sub panels: *i*–*iii*). The stations where distributed symmetrically with a constant spacing of 10 mm for all three wings, which corresponds to the following number of spanwise stations per wing: 37 at 5° sweep; 31 at 30° sweep; and 23 at 50° sweep. For each wing the suite of scanned chordwise contour lines were used to interpolate the 3D surface in the spanwise direction (Fig. 2A–C); *std*<0.05 mm (n = 3; we repeated the scan three times). To quantify the roughness of the wing we first removed the large-scale curvature as follows: the leading and trailing edge of the upper surface contour were assigned the same height by rotating each cross-sectional image. We then fitted the mean-chord line of a NACA 4-series airfoil through the upper surface contour, with an additional quartic term to capture leading edge curvature (we found this airfoil-based fit worked better than polynomial, Fourier, or circular fits). The roughness distributions (Fig. 2A–C) were then calculated relative to this smoothed airfoil-like mean shape.

### Wind tunnel measurements

In 2005 we used the Delft low-turbulence wind tunnel to study the aerodynamics of swift wings. We performed laminar-turbulent transition measurements, flow visualization, and lift and drag measurements, of which the visualization and forces have been described earlier [7]. The wind tunnel has a turbulence level of 0.025% at 40 ms^−1^ and 0.015% at 20 ms^−1^; for details and references see [7]. We measured boundary-layer laminar-turbulent transition locations on the upper surface of 3 wing pairs, with 5°, 30°, and 50° sweep, for all combinations of wind speeds (5–25 ms^−1^ in 5 ms^−1^ increments) and angles of attack (0°–18° with 4.5° increments) relevant for gliding swifts. For wind speed and angle of attack combinations at which swift wings stalled or deformed dramatically, transition measurements were impossible [7]. To test the effect of the primary-feather-like roughness on the extent of laminar flow, we built two model wings, one with and one without roughness. The wings are made out of carefully selected C-grain balsa wood strengthened by a carbon fiber spar and have a chord of 38.1 mm and a span of 360.0 mm. We approximated the airfoil of the swift hand wing using a thin airfoil with a small nose radius and sharp trailing edge; the maximal thickness was 2.8% (at 12.6–32.6% chord). To add camber we dried the wings in an oven after soaking them in water and fixing them on a circular mold using a bandage. The resulting camber was circular with a height of 4.5% chord at 50% chord. Swift roughness was simulated using a stack of thin strips of tape with a thickness of 0.05 mm each (TrimLine, ModelTechnics). Transition of the flow from laminar to turbulent was measured using an amplified stethoscope [29], [30] (Fig. 1B; Fig. S1).

We selected the stethoscope technique because it is particular effective for scanning transition location over the compliant, absorbent, heat sensitive and reflective rachises, barbs and barbules. The unique advantage of a stethoscope is that it can easily accommodate deformation by traversing the stethoscope by hand. Oil visualization is problematic because the porous wing absorbs fluorescent oil, as demonstrated by the dramatic effect of oil spills on bird feathers. Hot-wire probes are very sensitive to touch, and feather barbules and barbs smolder at the elevated operating temperatures (hence feather fletching is shaped using hot wires). Feathers also absorb the smoke drops used for particle image velocimetry (PIV), and are reflective (Fig. 1A), which deteriorates optical resolution in the approximately 1 mm thin boundary layer of swift wings.

The stethoscope can detect the signature broad-spectrum noise of turbulent pressure fluctuations and distinguish it from tonal noise and the silence of laminar flow (Fig. 1C,D). The stethoscope system consisted of a custom head (Fig S1A,B) mounted on a Brüel & Kjaer (B&K) microphone system, the latter comprising a microphone model 4134 (no 2060204), a preamplifier model 2619 (no. 805038), and power supply model 2801; the B&K system was connected to a Geloso amplifier system model G.1/2030. Timmer [31] verified that this system has an accuracy of 0.5–1.0% chord length for an airfoil with a chord length of 0.25 m, by direct comparison with infrared transition measurements in the same wind tunnel. This relative accuracy translates into a spatial accuracy of about 1.3–2.5 mm, which is 1–2 stethoscope diameters (Fig. S1) and represents an uncertainty of 3.5–7% chord length for the average swift wing in this study (36 mm wing chord: averaged for 5°, 30°, and 50° sweep). To illustrate how the stethoscope works we recorded two demonstration sequences (carried out faster than the actual measurement to keep the videos short). During the actual experiments, the stethoscope was kept stationary with respect to the wing.

The demonstration videos (Movie S1 and S2) were recorded with a JVC GR-DVL9700 digital video camera (recording to MiniDV-tapes). The MiniDV signal was converted to an avi file with Pinnacle V9 using the ‘Intel-Ligos Indeo Interactive 5_0 (IV50)’ video codec. The audio channel of these movies was used to calculate the spectrograms in Fig. 1C,D with the MATLAB spectrogram function using a Hamming window. Finally, the movies were converted to AVI using the MPEG-4 video codec and PCM (32 bit; 44100 Hz; 2822 kbps) audio codec of AVS4YOU Video Editor (version 6.2.1.222).

### Transition measurement procedure

The stethoscope was traversed along the surface of the wing from the leading edge towards the trailing edge until the sound changed into a broad-spectrum signal, which indicates transition to turbulent flow (see Fig. 1C,D and Movies S1 and S2). A photograph of each transition point on the wing, indicated by the tip of the stethoscope, was captured with a digital single lens reflex camera (NIKON D100 with AF Zoom-Nikkor 35–70 mm f/3.3–4.5 N, mounted on a tripod) focused on the top surface of the wing (Fig. 1B). All wings were spanwise sampled with an average spacing of about 7% wingspan to measure the chordwise transition distribution. All transition locations were photographed except for wing regions in which the boundary layer was either fully laminar or fully turbulent; these regions were recorded directly in the lab journal noting the photograph numbers that enclosed these regions. For 5° sweep, the average number of transition points recorded for the fully photographed sets is 16 (minimum 11, maximum 19), giving an average spacing between spanwise transition points of 6% wingspan. For 30° sweep the average number of transition points recorded for the fully photographed sets is 14 (minimum 11, maximum 18), giving an average spacing between points of 7% of the wingspan. For 50° sweep the average number of points for the fully photographed sets was 13 (minimum 9, maximum 18), giving an average spacing of 8% of the wingspan.

In some cases, we also found a distinct tone (1010 Hz) that initiates upstream of the transition point, illustrated in Fig. 1C. The most parsimonious explanation of that tone (not audible without stethoscope) is Tollmien-Schlichting instability, which initiates transition to turbulence by aggregating vorticity in a wave with an acoustic signature [10], [16]. The latter sound is routinely detected by microphones in the boundary layer [32], [33]. The smallest unstable wavelength *λ*_min_ of Tollmien-Schlichting instability is *λ*_min_/*L* = 6*δ*/*L*≈0.18 for a flat plate [10]. This value is similar to the measured value *λ*/*L* = *U*/*fL* = 0.28 (*f* = 1010 Hz at *U* = 10 ms^−1^ and *L* = 0.036 m). The predicted and measured wavelengths are of the same order of magnitude, and similar to the wing corrugation wavelength of roughly 0.1 chord length (Fig. 2A,C).

### Transition data processing

**Figure 3:**
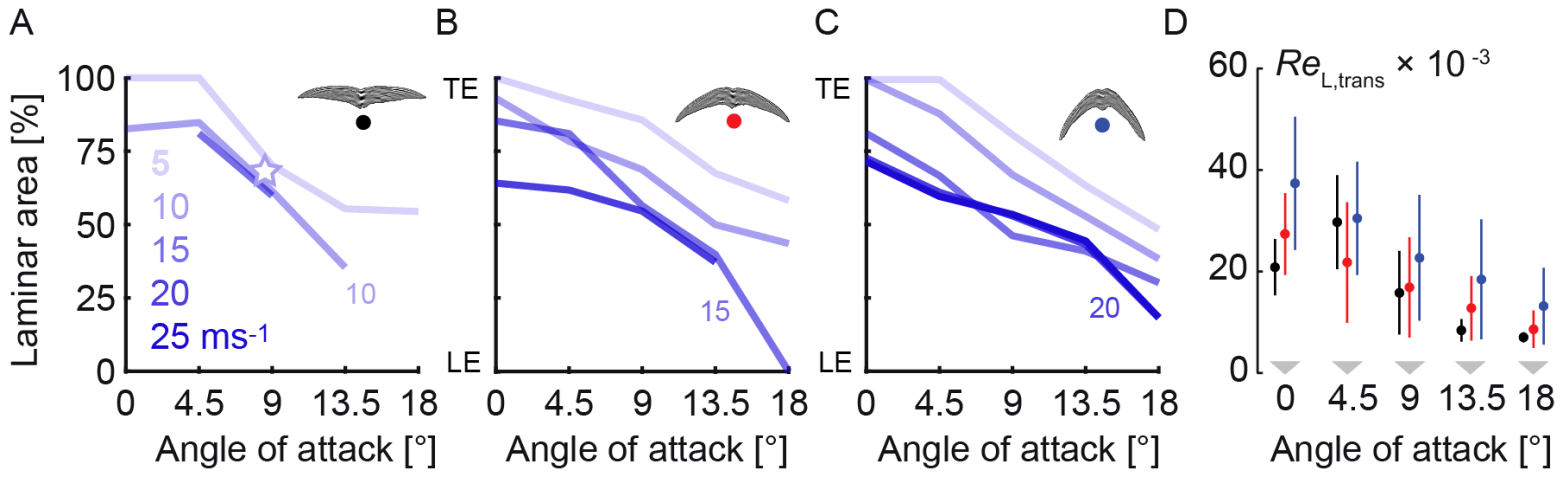
Increase of angle of attack and glide speed reduce the extent of laminar flow. (A,B,C) Transition remains aft of the most prominent feather roughness at angles of attack ≤9°. Laminar area in % corresponds to the span-averaged transition location: (A) 5° swept wings up to 15 ms^−1^ (black dot), the star shows interpolated laminar area for maximal glide performance at 8.5° and 8 ms^−1^; (B) 30° swept wings up to 20 ms^−1^ (red dot); (C) 50° swept wings up to 25 ms^−1^ (blue dot). The 20 and 25 ms^−1^ curves overlap in (C); LE: leading edge; TE: trailing edge. (D) The Reynolds number based on transition length (*L*_trans_) behind the leading edge is similar to the transition length behind an effective turbulator on a flat plate at 0°, for which Kraemer [34] found: *Re*_L,trans_ = 20,000. (black: 5°; red: 30°; blue: 50° sweep).

A custom program (scripted in MATLAB) was used to locate both wingtips, the tip of the stethoscope, and the leading and trailing edge at the spanwise location of the stethoscope. In concert with the logbook data, this resulted in spanwise distributions of laminar-turbulent transition location (shown in Fig. 2). The total laminar area (Fig. 3) was determined by integrating the transition location along the span (Fig. 2). We assumed that the leading edge corrugation induced the first perturbation and measured transition length with respect to it (Fig. 3D). We averaged *Re*_L,trans_ over wingspan and speed and excluded fully laminar stations for which it cannot be calculated. We determined Laminar area of the wings at the angles of attack that correspond to performance maxima by using linear interpolation, as follows. First the force and transition measurements for each wing and speed combination were linearly interpolated for angles of attack ranging from −1° to 20° (with steps of 0.1°) and speeds ranging from 5 ms^−1^ to 25 ms^−1^ (with steps of 0.1 ms^−1^). Points of the interpolation matrix that lay outside the measured range were not taken into account for further analysis. Using the interpolated force data we determined the angle of attack corresponding to equilibrium (straight glides) and maximal energy efficiency (turning glides) as a function of speed for each wing [7]. For these angle of attack – speed combinations, laminar area was linearly interpolated between the closest laminar area measurements (Fig. 3). The average extent and standard deviation of laminar flow area, at maximum glide performance, was first calculated for every sweep across speeds. Next, we calculated the Euclidian norm of the *std* of these means, and their respective *std*, across sweep.

MATLAB versions 2008a–2012b were used to process data and generate figures between 2010 and 2013.

## Results and Discussion

We measured laminar-turbulent transition over preserved swift wings in a low-turbulence wind tunnel, under conditions that replicate gliding flight (*Re* = 12,000–67,000). Using the stethoscope, we mapped the spanwise distribution of laminar flow (Fig. 2). At the speed (5 to 20 ms^−1^), sweep (5°–50°), and angle of attack combinations for which our lift and drag measurements [7] predict maximal glide distance and flight duration, we find that transition occurs well behind the turbulators on the hand wing. Transition is sensitive to small disturbances, such as differences in wing shape and preparation, which is reflected by the asymmetries in the distribution. The laminar area that we measured over freeze-dried swift wings represents therefore a reasonable underestimate for living swifts [7], [14]. To obtain an overview of the extent of laminar flow we integrate laminar area for all sweeps, speeds, and angles of attack (Fig. 3A–C). Such an integral representation for a whole wing averages over spanwise variation, which, in combination with the accuracy of the stethoscope, enables us to analyze the extent of laminar flow over the wing. The stethoscope can determine transition location with an accuracy of 3.5–7% of the average chord length (Methods). The perturbation caused by the thin tube can project transition in the boundary layer slightly forward [31], which reduces laminar flow. The measured fraction of laminar flow area shows that transition border moves toward the leading edge with increasing angle of attack and speed (plots in Fig. 3), in accord with boundary layer theory [10], [16]. Our measurements on swift wings show that transition to turbulence occurs at airspeeds as low as 5 ms^−1^, *Re* = 12,000, similar to 3D-printed hummingbird wings at 10° angle of attack [18]. The extent of laminar flow is, however, larger on real swift wings: approximately ½ chord, or more, at angles of attack up to 9° (n = 3; by combining results for 5°, 30°, and 50° sweep).

For airplanes large and small, the effect of surface roughness is to force the transition border forward on the wing [12], [26]. Turbulence occurs at a distance behind the turbulator, *L*_trans_, which the flow needs to transition into turbulence [16] (see Fig. S2). This distance between turbulator and transition point has been measured by Kraemer [34] for a flat plate at zero angle of attack at *Re* = 10,000–100,000 (based on full chord length). This *Re* range overlaps with the low Reynolds regime of swifts. For this range Kraemer found that the minimum Reynolds number based on transition length *L*_trans_ is approximately constant for fully effective turbulators: *Re*_L,trans_ = 68,000×*U*×*L*_trans_ = 20,000 (Fig. S2). This transition length is only 2% of the Reynolds number of a generic sailplane gliding at its optimal speed *Re* = 1,000,000 (*U* = 30 ms^−1^; *L* = 0.5 m; [12]). Transition length is therefore insignificant compared to chord for sailplanes – which is why sailplane design centers around transition delay [12]. In contrast, *Re*_L,trans_ is similar to the chord *Re*≈22,000 for swifts gliding at their optimal glide speed (*U*≈9 ms^−1^; *L* = 0.036 m). We calculate transition length for swift wings with respect to the wings' leading edge (close to the first bump) and find a transition length similar to Kraemer's 20,000 for a flat plate with a fully effective turbulator, Fig. 3D. Because the Reynolds number of swifts is low, the transition length is large compared to wing chord, which results in a laminar-flow wing despite feather roughness. This presumably enables swifts to exploit both the low friction drag of extensive laminar flow [10], [12], [16], [26] and the good stall characteristics [7] of low Reynolds number airfoils with turbulators [23]–[26], [35], [36].

**Figure 4:**
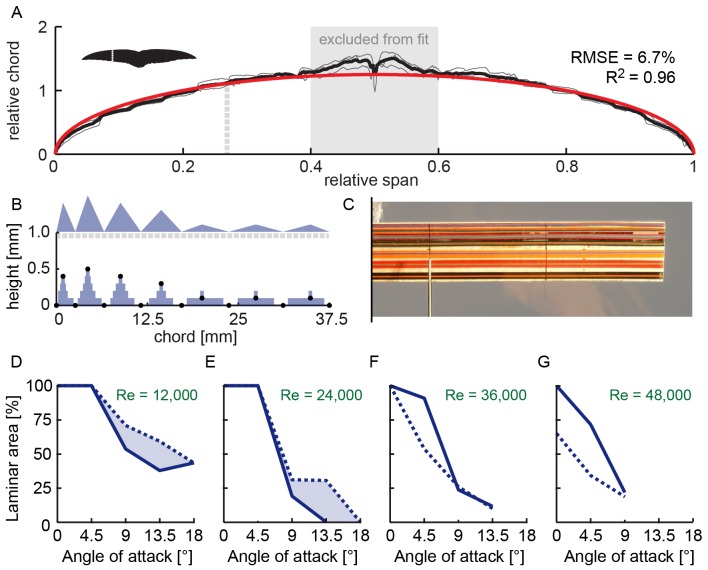
Simulated swift-like roughness extends laminar flow over model swift wings at Reynolds 12,000–24,000. (A) Fully extended swift wings are to a good approximation elliptical (n = 3; [7]), not unlike the Supermarine Spitfire. (B) Simulated 1.3% roughness of the swift hand wing using a distribution of thin tape stacks of various widths and heights similar to the roughness distribution in Fig. 2A, panel iii. The roughness is drawn to scale, with the y-axis expanded by a factor of 10 to reveal the shape of the actual roughness distribution on the model wing shown in (C) (cut at the symmetry plane for layout purposes). (D–G) Laminar area over the model wing, based on measurements at 4 equidistant stations that exclude the wingtip, for *Re* 12,000–48,000 (at higher speeds we did not measure transition at maximal incidence). Laminar flow is reduced by swift-like roughness at Reynolds numbers that correspond with glides beyond cruising speed. (Dashed line, roughness; continues line, smooth).

**Figure 5:**
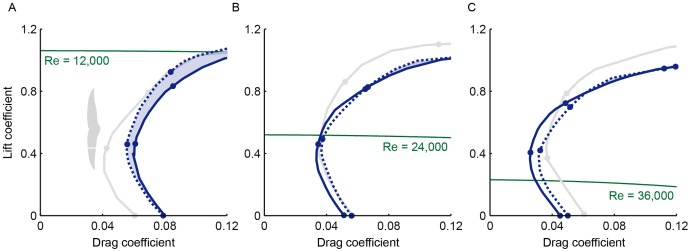
Swift-like roughness decreases drag below Reynolds 24,000 and increases drag beyond, during straight glides. Force measurements for actual swift wing are shown in gray at *Re* 12,000 (A), 24,000 (B), and 36,000 (C). The dots represent 0°, 4°.5°, 9°, 13.5° and 18° angle of attack, at which the laminar area has been determined (18° represents stall and is therefore not visible in A, B for model wings). The green curve corresponds to straight equilibrium glide conditions at that particular Reynolds number. (Dashed line, roughness; continues line, smooth).

To explore how swift-like roughness might influence laminar flow, and whether this improves performance, we tested models of fully extended hand wings (Fig. 4A) with and without turbulators. The roughness (Fig. 2A, panel iii) is simulated using stacks of 0.05 mm tape with a maximum height of 1.3% chord length (Fig. 4B, C). Swift-like roughness reduces laminar flow over model wings at Reynolds numbers beyond 24,000, Fig. 4F,G. Such a reduction is expected for Reynolds numbers closer to 100,000 [23]–[26], [35], [36]. Reynolds numbers beyond 24,000 are attained beyond cruise speeds of 10 ms^−1^ [7], since *Re* = 2,400 *U* for extended wings. In contrast, swift-like roughness promotes laminar flow during cruise at *Re* 24,000 down to 12,000. To determine how these differences in laminar flow area correspond with performance, we measured lift and drag using the same method published earlier for swift wings [7]. The lift and drag compare reasonably well with those of the fully extended swift wing, Fig. 5, when the lift coefficients are close to the values that are required for lift-weight equilibrium during straight glides [7]. The model wing with swift-like roughness has lower drag at *Re* 12,000–24,000 for lift coefficients close to equilibrium. For faster glides at *Re* 24,000 and up, roughness results in more drag. Reynolds number depends, however, not only on speed, but also on altitude. Swifts roost at altitudes of 1,500 m and flap glide at airspeeds of 9 ms^−1^ (over their wing) that minimize energy loss [7]. At 1,500 m altitude, Reynolds number falls to *Re* = 61,000×*U*×*L* for typical values of pressure and temperature lapse rate in the Standard Atmosphere, corresponding to *Re* 19,000. Swifts and swallows flying at even lower Reynolds numbers might benefit more from wing roughness. At higher Reynolds numbers, *Re* 24,000–48,000, swift-like roughness reduces laminar area and increases drag for lift coefficients close to equilibrium gliding. At these higher Reynolds numbers swifts typically sweep their wings backward to reduce drag [7]. The roughness measurement for swift wings (Fig. 2) suggests this reduces roughness height somewhat, which could be beneficial. The model wing measurements also suggest that swifts attain laminar flow over rough wings.

**Figure 6:**
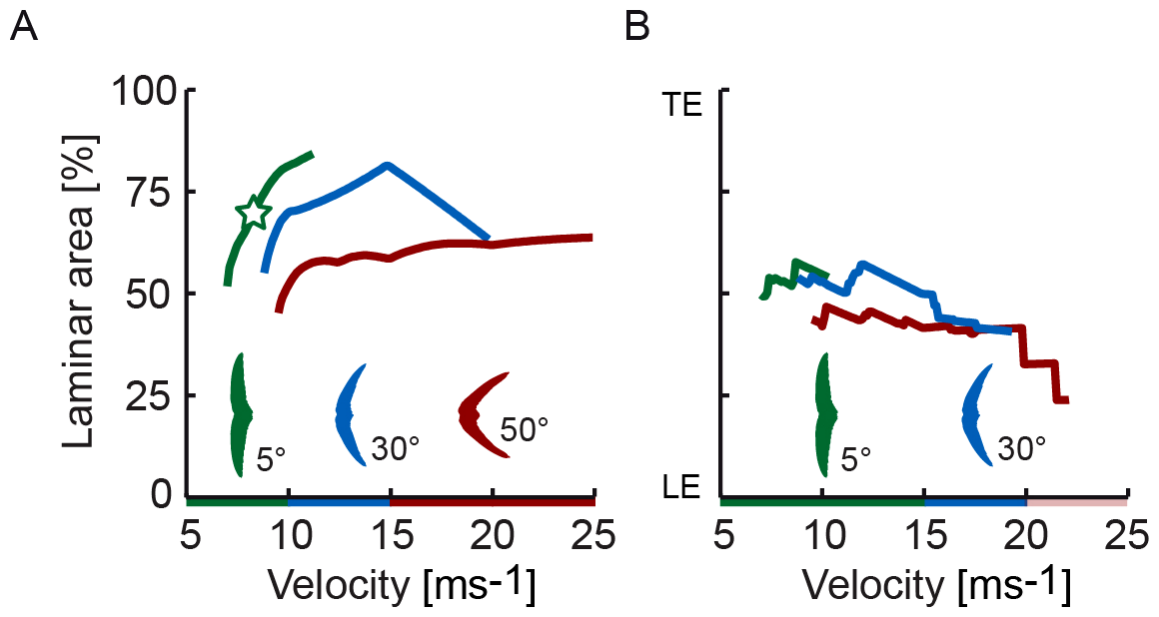
Angles of attack that minimize energy loss correspond with transition beyond ½ chord length. (ave 69% and *std* 13% for 5°, 30°, and 50° sweep combined) These angles of attack are inferred by calculating energy loss based on measured lift and drag [7]. (A) Laminar area for maximal glide distance and flight duration during straight flight. Fully extended wings (green) minimize energy loss up to ∼10 ms^−1^, but at higher speeds swept-back wings (blue, red) excel. The green star corresponds to a laminar area close to ¾ chord length (n = 1 wing pair) at minimal energy loss [7]. (B) Laminar area for maximum turn angle, which requires high lift to limit height loss, is about ½ chord length (ave 48% and *std* 11% for 5°, 30°, and 50° sweep combined). Fully extended wings (green) generate more lift, but at speeds beyond ∼15 ms^−1^ only swept wings (blue) can bear the load [7].

By interpolating laminar area for sweep, speed, and angle of attack combinations (Fig. 3) at which our lift and drag measurements predict minimal energy loss [7], we determined that the average extent of laminar flow over swift wings is 69% chord length (*std* 13%; n = 3) over 5°, 30°, and 50° sweep (Fig. 6A). At maximum turn performance (maximum turn angle per meter altitude loss) we find similarly that the average extent of laminar flow over swift wings is 48% chord length (*std* 11%; n = 3) over 5°, 30°, and 50° sweep (Fig. 6B). The pooled averages (three different wing pairs (n = 3) prepared at three different sweeps) show that, at maximum glide performance, laminar area extends beyond the most-perturbing feather valleys and ridges for a range of sweeps and speeds. At minimal energy loss during straight flight, which swifts achieve with fully extended wings gliding close to 9 ms^−1^ [7], [14], we find that transition occurs at ¾ chord length (n = 1; 5° sweep). This value is close to the average value of 67% (*std* 13%; n = 3) for efficient straight gliding across speeds. Taking the measurement uncertainty of 3.5–7% into account, the extent of laminar flow over aerodynamically rough wings, across speeds and wing sweeps at maximum glide performance, can compete with NASA's smooth laminar flow wings [37], [38] and high-performance sailplanes [12]. The rough swift wings analyzed here can generate laminar flow because the flow needs the distance from the leading to the trailing edge (wing chord) to develop into turbulence. This transit allows for unexpected degrees of freedom at the level of surface architecture. Thus small birds, such as swifts and swallows, can afford thicker protruding rachis, which are stiffer and stronger, without sacrificing aerodynamics. This aerodynamic niche at low Reynolds numbers might have provided small birds an evolutionary window to generate laminar flow and glide well despite their rudimentary wing surface architectures. This window is also available for the design of swift-sized micro air vehicles [39] that do not depend on the high-Reynolds-number paradigm – smooth surfaces – for efficiency.

## Supporting Information

Figure S1 **Stethoscope dimensions.** Dimensions of the stethoscope [29], [30] we used to listen to, and record, turbulent pressure fluctuations in the boundary layer. (A) The stethoscope consists of a 1.35 mm OD tube connected to a small acoustic chamber with a microphone. (B) Detailed sketch of the stethoscope showing its main dimensions (courtesy of Stefan Bernardy).
(JPG)

Figure S2 **Transition on a flat plate at low Reynolds number.** The Reynolds number based on transition length behind a wire turbulator on a flat plate as a function of chord Reynolds number, strip location, and strip thickness; adapted from Kraemer [34]. These results for a flat plate at zero angle of attack show that the minimum Reynolds number based on transition length behind the wire *L_trans_* = *X*_tr_–*X*_k_ is 20,000 for chord based Reynolds numbers ranging from 10,000 to 100,000. The figure was scanned from Schlichting [16]. The Reynolds numbers corresponding to fully turbulent flow are highlighted in Adobe Illustrator (CS6); these points have been digitized using ImageJ (Java Version 1.6.0_20 (32-bit)). The digitized points were used to calculate the average critical Reynolds number based on roughness height for the thick (*Re*_k_ = 430; *std* = 110) and thin (*Re*_k_ = 350; *std* = 60) wire. The results for the wire located at the leading edge (points *a* and *g*) were ignored because the values are difficult to obtain accurately; for points *f* and *h* no measurements have been reported in [16].
(JPG)

Movie S1 **Laminar-turbulent transition at maximum lift-drag ratio.** Video demonstration of the detection of laminar-turbulent transition with the stethoscope on a fully extended swift wing at 10 ms^−1^ attaining maximum lift to drag ratio. At some wing sections, a distinct tone can be heard at a frequency of about 1010 Hz.
(WMV)

Movie S2 **Laminar-turbulent transition at maximum lift.** Video demonstration of the detection of laminar-turbulent transition with the stethoscope on a fully extended swift wing at 10 ms^−1^ attaining maximum lift coefficient. At maximum lift the wing was partly stalled, resulting in light vibration, which complicated the use of the stethoscope. If the wing hits the stethoscope it can move a feather, which requires care during actual measurements. The original position of the feather was typically recovered by resetting the angle of attack and speed to zero and, if needed, careful preening of the feathers, and starting over.
(WMV)
