# Supplementary figures and images for: Gliding Swifts Attain Laminar Flow over Rough Wings
Source: PLoS One. 2014 Jun 25;9(6):e99901. doi: 10.1371/journal.pone.0099901 (PMC4070913; doi:10.1371/journal.pone.0099901)

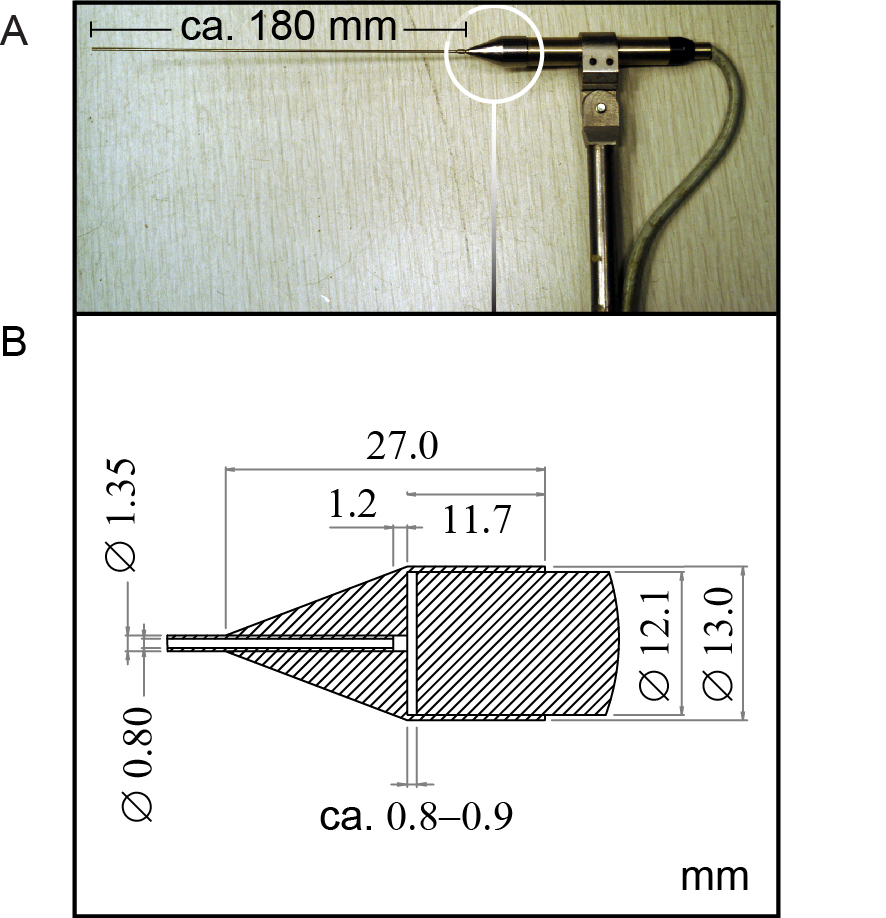

Supplement: Figure S1 — Stethoscope dimensions. Dimensions of the stethoscope [29], [30] we used to listen to, and record, turbulent pressure fluctuations in the boundary layer. (A) The stethoscope consists of a 1.35 mm OD tube connected to a small acoustic chamber with a microphone. (B) Detailed sketch of the stethoscope showing its main dimensions (courtesy of Stefan Bernardy). (JPG) [file pone.0099901.s001.jpg]

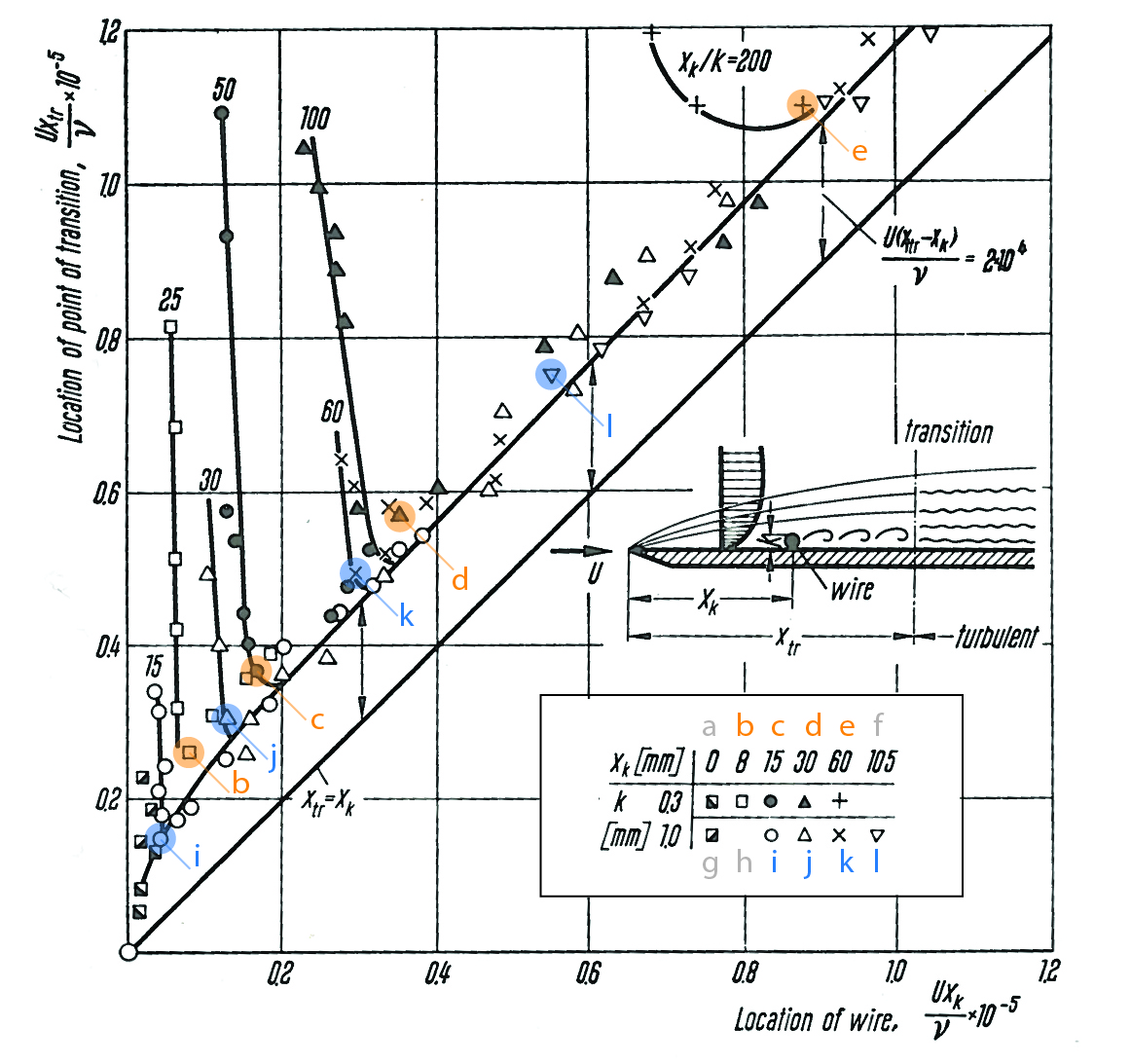

Supplement: Figure S2 — Transition on a flat plate at low Reynolds number. The Reynolds number based on transition length behind a wire turbulator on a flat plate as a function of chord Reynolds number, strip location, and strip thickness; adapted from Kraemer [34]. These results for a flat plate at zero angle of attack show that the minimum Reynolds number based on transition length behind the wire Ltrans = X tr–X k is 20,000 for chord based Reynolds numbers ranging from 10,000 to 100,000. The figure was scanned from Schlichting [16]. The Reynolds numbers corresponding to fully turbulent flow are highlighted in Adobe Illustrator (CS6); these points have been digitized using ImageJ (Java Version 1.6.0_20 (32-bit)). The digitized points were used to calculate the average critical Reynolds number based on roughness height for the thick (Re k = 430; std = 110) and thin (Re k = 350; std = 60) wire. The results for the wire located at the leading edge (points a and g) were ignored because the values are difficult to obtain accurately; for points f and h no measurements have been reported in [16]. (JPG) [file pone.0099901.s002.jpg]
